# Implementation of Nutrigenetics and Nutrigenomics Research and Training Activities for Developing Precision Nutrition Strategies in Malaysia

**DOI:** 10.3390/nu14235108

**Published:** 2022-12-01

**Authors:** Anto Cordelia T. A. Dhanapal, Ramatu Wuni, Eduard F. Ventura, Teh Kuan Chiet, Eddy S. G. Cheah, Annaletchumy Loganathan, Phoon Lee Quen, Mahenderan Appukutty, Mohd F. M. Noh, Ian Givens, Karani Santhanakrishnan Vimaleswaran

**Affiliations:** 1 Centre for Biomedical and Nutrition Research, Universiti Tunku Abdul Rahman, Jalan Universiti, Bandar Barat, Kampar 31900, Malaysia; 2 Hugh Sinclair Unit of Human Nutrition, Department of Food and Nutritional Sciences and Institute for Cardiovascular and Metabolic Research (ICMR), University of Reading, Reading RG6 6DZ, UK; 3 Centre for Community Health Studies (ReaCH), Faculty of Health Sciences, Universiti Kebangsaan Malaysia, Kuala Lumpur 50300, Malaysia; 4 Faculty of Sports Science and Recreation, Universiti Teknologi MARA, Shah Alam 40450, Malaysia; 5 Nutrition Society of Malaysia, Jalan PJS 1/48 off Jalan Klang Lama, Petaling Jaya 46150, Malaysia; 6 Institute for Medical Research, National Institutes of Health, Jalan Setia Murni U13/52, Shah Alam 40170, Malaysia; 7 Institute for Food, Nutrition and Health (IFNH), University of Reading, Reading RG6 6AH, UK

**Keywords:** precision nutrition, malnutrition, nutrigenetics, nutrigenomics, nutri-epigenetics, nutrition, artificial intelligence

## Abstract

Nutritional epidemiological studies show a triple burden of malnutrition with disparate prevalence across the coexisting ethnicities in Malaysia. To tackle malnutrition and related conditions in Malaysia, research in the new and evolving field of nutrigenetics and nutrigenomics is essential. As part of the Gene-Nutrient Interactions (GeNuIne) Collaboration, the Nutrigenetics and Nutrigenomics Research and Training Unit (N^2^RTU) aims to solve the malnutrition paradox. This review discusses and presents a conceptual framework that shows the pathway to implementing and strengthening precision nutrition strategies in Malaysia. The framework is divided into: (1) Research and (2) Training and Resource Development. The first arm collects data from genetics, genomics, transcriptomics, metabolomics, gut microbiome, and phenotypic and lifestyle factors to conduct nutrigenetic, nutrigenomic, and nutri-epigenetic studies. The second arm is focused on training and resource development to improve the capacity of the stakeholders (academia, healthcare professionals, policymakers, and the food industry) to utilise the findings generated by research in their respective fields. Finally, the N^2^RTU framework foresees its applications in artificial intelligence and the implementation of precision nutrition through the action of stakeholders.

## 1. Introduction

Nutritional epidemiological studies in Malaysia show a triple burden of malnutrition: undernutrition, overnutrition, and micronutrient deficiencies; approximately 20% of women and 15% of men are affected by obesity, while anaemia affects 32% of women of reproductive age [1]. This issue is highlighted in Malaysia’s National Plan of Action for Nutrition III, 2016–2025, which includes standard nutrition guidelines for various targeted groups [2]. The available data from the Global Health Observatory and the 2019 National Health and Morbidity Survey (NHMS) show a high prevalence of low birthweight, stunting in children under 5 years of age, and cardiometabolic diseases [3,4,5,6,7,8]. The NHMS suggests that the increased prevalence may be due to unhealthy food consumption trends among Malaysians [8], and the Institute for Health Metrics and Evaluation reported that among the ten most prominent risk factors that influenced disability-adjusted life years in 2019, six are related to nutrition: high blood pressure, dietary risks, high body mass index, hyperglycaemia, hyperlipidaemia, and malnutrition [9].

The nutrigenetic, nutrigenomic, and nutri-epigenetic research-based approach stands out as one of the domains of precision nutrition that can build up progress towards personalised nutrition interventions [10]. It provides opportunities to improve the understanding on the influence of nutritional and environmental factors on genetic susceptibility to malnutrition-related diseases [11,12,13,14]. The triple burden of malnutrition might vary across the genetic admixture in the sub-ethnic groups of Peninsular Malaysia [15,16] in relation to the following factors: malnutrition during pregnancy, maternity, and early stages of life, as well as intra-household nutritional differences between children and adults [17]; the vulnerability to metabolic abnormalities being passed down across generations [11]; and the exposure to obesogenic environment, food insecurity, and ultra-processed foods [18,19,20,21,22]. How these variables interact throughout the genetic admixture of Malaysians remains unknown and is obscured by the public health all-encompassing approach that precludes nutrigenetics, nutrigenomics, and nutri-epigenetics research in decision-making. In addition, a number of ethnicities and sub-ethnic groups coexist in Malaysia: Malay, Chinese, Indian, and Orang Asli; research has shown significant differences across these ethnicities in the prevalence of metabolic syndrome [16,23]. Furthermore, the nutritional requirements and metabolic responses to food vary among ethnicities due to genetic heterogeneity, and tailoring dietary advice to sub-groups of populations is more likely to address malnutrition in multi-ethnic populations such as Malaysians than conventional approaches alone [24].

The current gaps in knowledge regarding personalised nutrition in Malaysia have been highlighted as a challenge to tackling non-communicable diseases (NCDs) [25]. The nutrigenetic, nutrigenomic, and nutri-epigenetic-based approach and the “one-size-fits-all” public health approach complement each other; integrating both approaches is necessary to meet the needs of sub-populations, and this will require training of researchers and stakeholders. To this end, the Nutrigenetics and Nutrigenomics Research and Training Unit (N^2^RTU) was implemented in Malaysia in February 2022 as part of the Gene–Nutrient Interactions (GeNuIne) Collaboration [26] with funding from the British Council, supported by the Malaysian Industry-Government Group for High Technology (MIGHT).

The project is led by the University of Reading, UK and Universiti Tunku Abdul Rahman (UTAR) in Malaysia, and the primary objective is to facilitate knowledge and technology transfer by enabling collaboration between researchers from the two universities alongside the Nutrition, Metabolism & Cardiovascular Research Centre (NMCRC) of the Institute for Medical Research, National Institutes of Health (NIH), Ministry of Health Malaysia (MOH), and Nutrition Society of Malaysia (NSM) as associate partners. By bringing together experts from different fields, the N^2^RTU provides a platform for research and training in the new and evolving field of nutrigenetics and nutrigenomics. The evidence generated will be used by experts from the Institute for Food, Nutrition and Health at the University of Reading and the Centre for Biomedical and Nutrition Research (CBNR) in UTAR, as well as from the MOH and NSM, to develop dietary recommendations that are tailored to specific population subgroups to help reduce the burden of malnutrition and cardiometabolic diseases.

**Figure 1:**
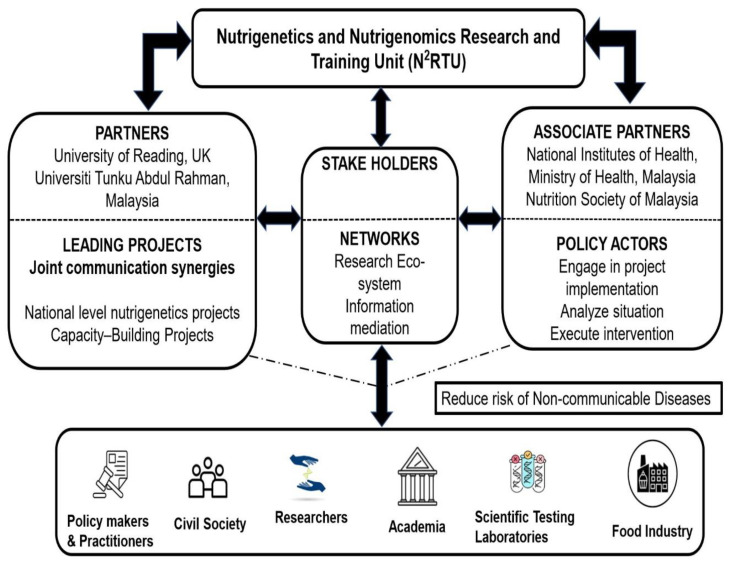
Inferential linkage between N^2^RTU partnerships and relevant stakeholders.

Different frameworks have emerged to: evaluate the evidence generated from nutrigenetic practice and research [27]; investigate precision nutrition [28]; apply nutrigenetics in personal, population, and planetary health [29]; guide health care providers in the practice of personalised nutrition [30]; collect genetic and environmental data from the Malaysian population by the `Malaysian Node of the Human Variome Project’ (MyHVP) [31]; predict the perceived risks and benefits of stakeholders towards the implementation of nutrigenetics in Malaysia [32]; and represent the aetiological complexity of obesity in a dynamic framework that includes genetics [33]. Moreover, stakeholders in Malaysia have shown interest in adopting nutrigenetics in the decision-making for health and wellbeing [32]. Nevertheless, there are no frameworks that guide the process of implementing a research and training unit focusing on nutrigenetics and nutrigenomics in developing the personalised nutrition approach, particularly at a national level in Malaysia. In this article, we will develop a framework, review what is necessary to implement N^2^RTU in Malaysia, and reflect on how it can translate into valuable knowledge for various stakeholders. Figure 1 gives an overview of the entire project.

Figure 1 demonstrates the role of the partners and associate partners of N^2^RTU and their linkages with the different stakeholders. Aiming to facilitate nutrigenetics and nutrigenomics research in Malaysia, the partners identify the capability needs and data gaps in the corresponding areas of nutritional genomics; lead the project activities and translate the capability needs and possible solutions into research needs; and explore funding opportunities and synergies between different funding bodies. The associate partners analyse and integrate the research findings for inclusion under national nutrition policies and try to implement nutrition intervention for the targeted population and sub-groups. The stakeholders (practitioners, namely clinicians and healthcare professionals, policy makers, research and academia, scientific and food industry and the civil society) will be involved in the planning and execution of nutrigenetics, nutrigenomics and precision nutrition research and serve as the main source of data collection. The views and ideas of the public will be integrated into the action plan as a holistic approach to prevent the incidence of non-communicable diseases (NCDs) in Malaysia.

## 2. N^2^RTU Framework Implementation Overview

**Figure 2:**
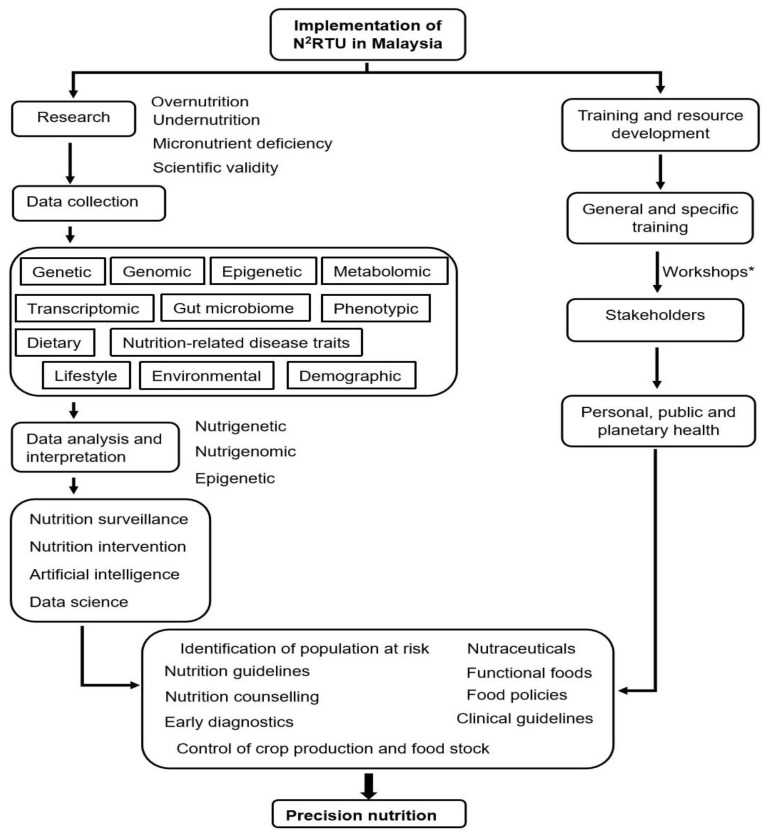
A framework showing the implementation of a Nutrigenetic and Nutrigenomic Research and Training Unit (N^2^RTU) in Malaysia and how it will contribute to tackling malnutrition and cardiometabolic diseases through precision nutrition. * Details of the workshops are given in Supplementary Table S1.

The path to achieving precision nutrition in Malaysia is shown in Figure 2, where the framework elucidates the pathway via research and capacity-building in nutrigenetics, nutrigenomics, and nutri-epigenetics. The ongoing training workshops for academics and other stakeholders will improve the understanding of research methods and the many applications of genetics, genomics, and epigenetics in nutrition. The collaborative use of the knowledge generated will help to tailor personalised nutrition at the population level, improve early identification of groups at risk of diet-related diseases, and aid in the treatment of diseases. The implementation of N^2^RTU in Malaysia seeks to influence the triple burden of malnutrition through improved understanding on how the environment and nutrition impact genetic vulnerability to disease and the mapping of gene–nutrient interactions in Malaysia. The knowledge gained from the research performed will be of value to stakeholders in their respective disciplines (academia, healthcare, policymaking, and the food industry). Finally, the use of artificial intelligence in integrating the data generated to aid in risk prediction is to be explored. The role of N^2^RTU in research, training, capacity-building, and the subsequent use of the output knowledge by stakeholders and in artificial intelligence in a collective effort to combat malnutrition in Malaysia will be described in the following three sections.

## 3. Implementing a Nutrigenetics and Nutrigenomics Research Unit

The Nutrigenetics and Nutrigenomics Research Unit is made up of a professional multidisciplinary research team comprising nutrition and genetic experts, phlebotomists, and laboratory technicians. This initiative covers different phases of research including study design, data collection, laboratory work, statistical analysis, and interpretation. In this way, genetic, metabolomic, epigenetic, gut microbiome, malnutrition-related disease traits, demographics, lifestyle factors, dietary and phenotypic data are collected not only as research outputs, but also as inputs for a database to be analysed and interpreted, thus generating nutrigenetic, nutrigenomic, and nutri-epigenetic knowledge among the Malaysian population. Machine learning and artificial intelligence will be employed to integrate the data from nutrigenetics, nutrigenomics, epigenetics, metabolomics, and gut microbiome. Machine learning has the potential to integrate omics data through the extraction of patterns in large datasets and clustering to generate predictive models and algorithms [34]. One of the machine learning methods is supervised machine learning, which is aimed at generating models that can accurately predict the data [35]. Through data mining, patterns and key indicators are extracted which are then used to find correlations within the various data groups (nutrigenetics, nutrigenomics, nutri-epigenetics, metabolomics and gut microbiota) [34]. Machine learning also involves supervised multivariate analysis, an example of which is partial least squares regression (PLSR), which works by identifying variables in groups of data which are most associated with the outcome of interest, thereby reducing the number of predictor variables [36,37]. In a study to determine the factors involved in insulin sensitivity, PLSR was successfully used to integrate data from diet, physical activity, gut microbiome, metabolomics, and gene expression in subcutaneous adipose tissue [36].

**Figure 3:**
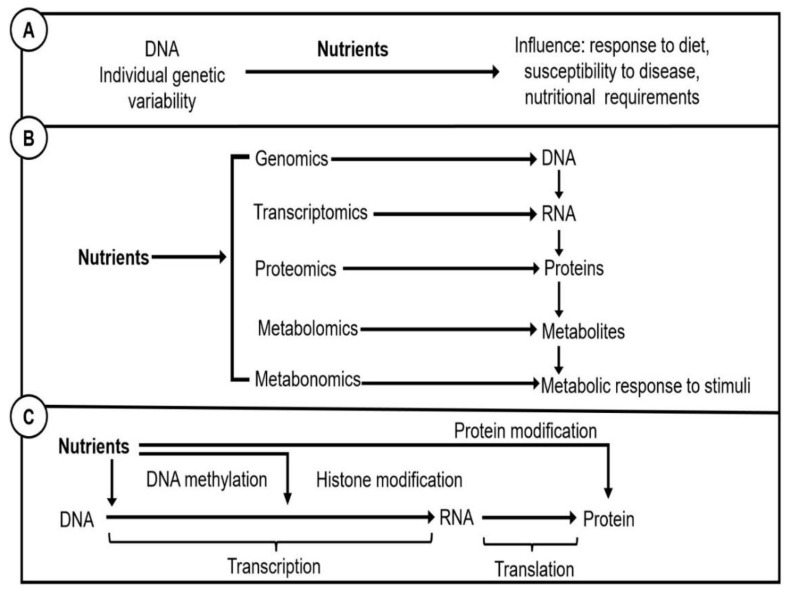
Representation of: (**A**), Nutrigenetics; (**B**), Nutrigenomics; (**C**), Nutri-epigenetics.

Figure 3 explains what these terms represent. Nutrigenetics is the study of the interactions between nutrition and genetic differences, often known as single nucleotide polymorphisms (SNPs), which can be aggregated into a genetic risk score (GRS) [26,38]. Genetic variations may influence protein synthesis and functions, thus altering dietary needs and metabolism and influencing a person’s risk of developing chronic diseases [10,38,39,40,41]. Nutrigenomics is the study of how nutrients and diet impact on genes, proteins, and metabolites as part of the omic sciences. Finally, nutri-epigenetics is the study of the ways by which foods affect gene expression; the path from genes to protein synthesis that does not involve DNA sequence changes.

To tackle undernutrition, overnutrition, and micronutrient deficiencies, the lessons learned can be retrieved from studies conducted in similar contexts in order to enhance the scientific validity of the research performed. The past implementation of N^2^RTU by the GeNuIne Collaboration in a variety of contexts gives a background knowledge that can be extrapolated to other research units [40,41,42,43,44,45,46,47,48,49,50,51,52,53]. The strengths identified from previous nutrigenetic studies include a multidisciplinary research approach and the validity of methods used for dietary data collection, such as culturally adapted semi-structured food frequency questionnaires. In addition, future considerations include the use of genetic risk scores rather than individual SNPs, large sample sizes, and strategies to improve follow-up. The inclusion of data on total energy and macronutrient intake, data on specific types of food or micronutrients, as well as accounting for various confounding factors in the statistical analysis, need to be taken into consideration [26,39,51,54,55]. To this end, training workshops will include ethics protocols, software for data collection and analysis, and research methods to enhance reproducibility (see Supplementary Table S1).

The results of the research will pave the way for stratification of the Malaysian population based on genetic and metabolic profiles so that precise dietary interventions can be implemented for sub-groups of the population with similar genetic or metabolic profiles [26,56]. This will be achieved through collaboration of the partners and associate partners of the N^2^RTU with academics from other Malaysian institutions. Results of nutrigenetic studies by the GeNuIne Collaboration indicate that individuals with a genetic predisposition to cardiometabolic traits such as obesity, dyslipidaemia, and hyperglycaemia are responsive to particular nutrients in an ethnic-specific manner [26,39,44,55,57,58] but the translation of these findings into clinical practice requires the joint effort of different stakeholders, and the N^2^RTU is well placed to achieve this. Studies have also shown that baseline metabolic profiles can predict responses to nutritional interventions [59,60]. Hence, the findings of the research will be used to devise reference values of metabolites for the Malaysian population to facilitate the use of metabolites as biomarkers in the development of precision nutrition in Malaysia [56,59]. Furthermore, by linking phenotypic data with metabolic profiles, researchers at the N^2^RTU will be able to identify particular phenotypes in Malaysians that are linked to adverse metabolic profiles, allowing for further stratification of sub-groups of the population for targeted nutrition interventions.

## 4. Implementing Nutrigenetics and Nutrigenomics Training for Stakeholders for Precision Nutrition in Malaysia

Precision nutrition is based on general nutrition, individual nutrition based on phenotypes, and genetic-based recommendations [10]. Currently, malnutrition in Malaysia is being addressed from a traditional triple-burden approach resulting in continued negative trends of stunting, micronutrient deficiencies, obesity, and other diet-related NCDs [61]. Notwithstanding, current recommendations do not incorporate evidence from genetic-based sciences. Private hospitals in major cities like Kuala Lumpur and Penang are the only ones with genetic counsellors supported by the MOH. Furthermore, the ratio of medical geneticists in the Malaysian population was 1 in every 3 million in 2013, providing counselling services only for prenatal, reproductive, paediatric, hereditary, and adult-onset genetic disorders [62]. Nutrigenetics, nutrigenomics, and nutri-epigenetics are powerful tools that have not yet been used in the Malaysia’s health system. These tools combine knowledge from the fields of nutritional epidemiology, biochemistry, bioinformatics, and emerging genomic technologies. The sections that follow will describe how stakeholders have approached malnutrition and what training is needed with regard to nutrigenetics, nutrigenomics, and nutri-epigenetics (see Supplementary Table S1).

### 4.1. Academia

The human genome variation map aims to provide data on genetic variations and mutations among the ethnic groups in Malaysia to further explore the interaction of lifestyle factors in genetic susceptibility to disease as part of the Malaysian Sizing (MYSIZE) survey project [63]. Nevertheless, the ‘MyHVP Database’ has only gathered data from genetic variations and mutations among the Malay population [31], leaving a number of ethnicities and sub-ethnicities unrepresented. The ‘Malaysian gene-environment multiplier hypothesis’ suggests that the interaction between the genetic evolution influenced by the feast and famine cycles and the current exposure to westernised lifestyles is leading to an increased genetic susceptibility to store fat and develop metabolic diseases [64]. Nutrigenetic research in the Malaysian population has investigated interactions between variants of insulin like growth factor 1 (*IGF-1*) gene and dietary acid load on bone resorption [65]; golgin A7 family member B (*GOLGA7B*) gene and a high glycaemic index diet on acne vulgaris [66]; vitamin D receptor (*VDR*) and group-specific component (*GC*) genes and maternal vitamin D status on birth anthropometric measures [67]; adrenoceptor beta 2 (*ADRB2*) gene and dietary fat on diabetes traits [68]; type 2 diabetes (T2D)-related genetic risk score of 62 SNPs and a number of environmental factors on T2D traits [69]; and major histocompatibility complex, class II, DR beta 1 (*HLA-DRB1*) shared epitope sequence and smoking on rheumatoid arthritis [70]. These studies suggest that there is a growing need and interest by the scientific community to investigate the interactions between genetic susceptibility and lifestyle. Due to inadequate scientific data on mechanistic role and interaction between diet or nutrient intake, biomolecules, and genes for personalised management of risk and control for diet-related NCDs, nutrition and health research priority in Malaysia for the 12th Malaysia Plan (2021–2025) had suggested research topics to be investigated on nutrigenomics and metabolomics, which include the identification of new nutritional biomarkers and development of nutrigenomics-based personalised nutrition intervention programmes for diet-related risk and control for the Malaysian population [71,72]. Moreover, it is necessary for academicians to look into providing education on nutrigenetics, nutrigenomics, and nutri-epigenetics during tertiary education and providing basic and latest knowledge and understanding on this topic to future researchers and healthcare professionals (HCPs), which would aid in building the capacity of the stakeholders.

Supplementary Table S1 shows details of the training workshops for academia, healthcare professionals (HCPs), policy makers, and the food industry.

### 4.2. Healthcare Professionals (HCPs)

The current approaches to malnutrition by public health professionals are based on the socioeconomic and phenotypic contexts, using data such as anthropometric indices and biochemical data [61]. HCPs from the public sector do not provide genetic-based nutrition counselling as their clinical guidelines have not incorporated evidence from this field. This is an option only accessible via the private health sector [73], and the current applications may be questionable as research among the Malaysian population remains scarce. Nutritional interventions can be tailored by HCPs to take into account genetic predisposition to diseases, effective population-specific nutrition interventions, and genetic variants determining dietary preference and metabolites, as well as phenotypes and intermediate metabolites [12]. Research has shown the effectiveness of nutrigenomic interventions over conventional nutritional interventions in obesity management [74], enabling identification of population subgroups who are more susceptible to changes due to caloric restriction [14].

HCPs tend to lack expertise and confidence when it comes to selecting and interpreting nutrigenetic tests [75]; thus, knowledge of nutrigenetics is essential to safeguard patients against the risks of direct-to-consumer marketing of nutrigenetic testing and prevent the use of fraudulent tests, deceptive claims, personalised dietary counselling, and untested dietary supplements. Furthermore, training is essential to appropriately translate the results from nutrigenetic tests to clinical practice [75]. It is important to note that a nutrigenomic care map has already been developed [30], providing a framework for HCPs to meet their training needs and gain confidence.

### 4.3. Policymakers

Malaysia has developed voluntary dietary policies to reduce the burden of malnutrition in all forms, with the exception of the forced tax on sugar-sweetened beverages to reduce sugar intake and reduce subsidy for cooking oils by 20%. To reduce obesity and NCDs, the policies seek commitment from the food industry, and to tackle maternal nutrition there are systems for the nutritional surveillance of gestational weight gain and micronutrient deficiencies which have been addressed with universal salt iodisation (implemented in January 2021), wheat flour fortification (implemented in July 2021), maternal nutrition counselling during pregnancy to tackle anaemia, and other measures such as extending maternal leave from 60 to 98 days to improve breastfeeding rates. Several programmes have been established to assist malnourished children, including the ‘rehabilitation programme for malnourished children’ that provides food baskets; the School Supplementary Feeding Programme (SSFP) that provides a free meal to primary school children from poor families, which ensures one-quarter to one-third of the Recommended Nutrient Intakes (RNI) for energy and protein; and the ‘School Milk Programme’ that supplies free milk daily and the ‘Nutritious Meal Programme’ or the ‘Healthy Food Canteen Management Guide’ to ensure school canteens provide healthy food [76]. To reduce childhood obesity and related diseases later in life, the “Circular of the Secretary-General of the Ministry of Housing and Local Government No. 4/2012 Guidelines for the Enforcement of the Prohibition of the Sale of Food and Beverages Outside School Fences by Local Authorities” prohibits the selling of processed products rich in salt, artificial flavourings and colourings, alcohols, sweets, and chocolates within 40 metres from school perimeters [77].

From a nutrigenomic standpoint, food and nutrition policies that focus on primary prevention have the potential to lower disease incidence before the crucial changes in gene expression occur. As a result, science-based treatments aimed at people from varied communities at higher risk of disease will not be undermined if food and nutrition regulations take into consideration the findings of nutrigenetic, nutrigenomic, and nutri-epigenetic investigations. Thus, knowledge of genetic variations and how they influence responsiveness to certain diets with regard to disease risk will help policymakers to tailor nutritional advice to those at increased risk [78], as well as implement strategies to prevent disorders in future generations [11]. Moreover, the need to regulate nutrigenetic services to avoid potential harm linked to unsubstantiated tests and health claims has been highlighted [79]. Hence, the training offered by the N^2^RTU is crucial in the development of regulatory guidelines to safeguard the public while tackling nutrition-related NCDs.

### 4.4. Food Industry

To tackle the burden of malnutrition via the food industry, a number of actions have been implemented. For example, the ‘Healthier Choice Logo’ (HCL) is a voluntary option to be used by the food industry in the labelling of products in order to give advice to consumers, and a zero goods and services tax (GST) has been implemented for wholegrain products with HCL [80]. In 2021, the government imposed mandatory declaration of total sugars and sodium in all food products at all stages and four types of fatty acids (saturated, monounsaturated, polyunsaturated, and trans fatty acids) in selected foods to help reduce obesity and NCDs and improve household finances [81]. However, the intake of vegetable oils (palm and palm kernel oil), sugars and sweeteners, and animal products is prominent in Malaysia’s nutrition transition and remains high [82]. Moreover, six states (Kedah, Penang, Perak, Pahang, Terengganu, and Kelantan) out of thirteen states and three Federal territories do not meet the recommended minimum iodine levels, and only about 50% of children meet the recommended dietary intake of vitamin C and thiamine, while only 35% meet the requirements for calcium intake, indicating the need for food fortification [61].

From the standpoint of the food sector, industrial revolution predicts that if enough data is collected from individuals, new technologies such as machine learning and blockchain will facilitate the development of personalised diets [83]. As a result of precision nutrition research, the food industry has produced gluten-free diets for people with coeliac disease, probiotics for individuals with lactose intolerance, phenylalanine-restricted diets for those with phenylketonuria, and galactose-free products for patients with galactosaemia [84]. Research evidence from the N^2^RTU will be invaluable to the Malaysian food industry in several ways: reformulation of existing products, development of nutraceuticals and functional foods, integration of functional bioactive compounds, identification of the population subgroups in need of fortification, and validation of health benefits of products [85,86]. In addition, the N^2^RTU will bring together HCPs from different backgrounds, thereby fostering the public-private partnerships that are necessary for the creation of new public health policies and for the reformulation of existing policies to tackle malnutrition and related diseases.

## 5. Future Implications of Information Systems and N^2^RTU in Artificial Intelligence

Since the 1980s, nutrition surveillance has been in place to keep track of the nutritional status of the Malaysian population. The ‘Nutrition Supervision Programme’ is a tool for developing and re-evaluating nutrition policies and programmes and obtaining data from the peripheral services to feed the Health Management Information System (HMIS) [87]. The HMIS works at national, regional, and district levels, and decision-making is based on the health data collected by this system [88]. However, the HMIS does not integrate data from the private sector, and hence does not reflect data collected from private health centres or data on genetic susceptibility to disease, nutrigenetics, nutrigenomics, or nutri-epigenetics. The ‘MyHVP Database’ provides information on genetic variations and mutations [31] that could be incorporated into the HMIS. New methods of data collection could help to incorporate data from multiple sources, thereby improving the statistical power and precision of measurement [89]. Research suggests that the use of technologies such as smartphones and microfluidics-based lab-on-a-chip platforms for quick testing can gather data and feed it into information systems effectively [90]. In addition, nutritional epidemiology can use current technology breakthroughs such as ‘Big Data’ and ‘Machine Learning’ as a groundbreaking analytical resource for dietary measurement and assessment and as a potential tool to model and deduce the complexity of dietary intake and its implication on health [89,91,92]. A system that employs data mining could predict metabolic responses to diet via the integration of data derived from genetics, microbiome, blood tests, anthropometrics, lifestyle factors, and diet into a single platform [88,91,93].

## 6. Conclusions

Current strategies for tackling malnutrition in Malaysia do not consider data from nutrigenetics, nutrigenomics, or nutri-epigenetics. The different stakeholders involved in tackling malnutrition require training and evidence from nutrigenetic and nutrigenomic research to guide and customise their decisions. The genetic admixture, exposure to lifestyle factors, and the persistence of malnutrition warrant further investigation. Although several studies have been conducted to address malnutrition, including cross-sectional, longitudinal, and interventional studies, the success of the current all-encompassing approach is blurred by the unexplained genetic susceptibility to disease, which might differ among Malaysia’s diverse ethnic groups. To this end, this paper has proposed the first framework for nutrigenetics and nutrigenomics research and training for academia, policymakers, HCPs, and the food industry in Malaysia. Furthermore, it suggests a data-driven approach based on artificial intelligence, utilising a centralised database that considers all drivers of malnutrition, including genetic susceptibilities across the Malaysian population, with emphasis on the most vulnerable groups. Future research should consider the unification of information systems used in Malaysia (such as the HMIS and the ‘MyHVP Database’) and the integration of knowledge generated from nutrigenetic, nutrigenomic, and nutri-epigenetic research as well as partnership working between public and private sector organisations in order to improve the health and wellbeing of the population.

## Data Availability

The study did not report any data.

## Supplementary Materials

The following supporting information can be downloaded at: https://www.mdpi.com/article/10.3390/nu14235108/s1, Table S1: Workshops for stakeholders.
