# Supplementary material for: Implementation of Nutrigenetics and Nutrigenomics Research and Training Activities for Developing Precision Nutrition Strategies in Malaysia
Source: Nutrients. 2022 Dec 1;14(23):5108. doi: 10.3390/nu14235108 (PMC9740135; doi:10.3390/nu14235108)
Supplement: Supplementary file 1 [file nutrients-14-05108-s001.zip › nutrients-1974252-supplementary.pdf]

Supplementary Materials

Table S1. Workshops for stakeholders.

Stakeholders	Workshops	Objectives	Results
**Academia**	- Research methods in nutrigenetics, nutrigenomics, and nutri-epigenetics<br>- Laboratory theories and practicums<br>- Software theories and practicums<br>- How to work in a multidisciplinary research environment<br>- Ethics and data protection	Learn statistical analysis; methodological advantages and disadvantages; laboratory techniques and software; improve collaboration among co-workers in multidisciplinary environments; ethical considerations and data protection measures.	- Improved ability to conduct nutrigenetics, nutrigenomics and nutri-epigenetics research<br>- Integration of nutrigenetics, nutrigenomics and nutri-epigenetics into the education curriculum.
**HCPs**	- Introduction to nutrigenetics, nutrigenomics, nutri-epigenetics, and precision nutrition<br>- Risks of direct-to-customer tests (DCTs) and how to safeguard patients from unreliable DCTs<br>- Reliable sources of information for genetic-based nutrition counselling<br>- Examples of genetic-based nutrition counselling<br>- Ethics and data protection	Learn the essentials of nutrigenetics, nutri-epigenetics, nutrigenomics, and precision nutrition; knowledge on choice of DCTs for specific patients; knowledge on nutrition counselling based on DCT results; identify reliable sources of information; how to translate evidence into practice; ethical considerations and data protection measures	- Improved ability to utilize data from nutrigenetics, nutrigenomics and nutri-epigenetics in clinical practice to promote health and reduce malnutrition-related diseases.
**Policymakers**	- Introduction to nutrigenetics, nutrigenomics, nutri-epigenetics, and precision nutrition<br>- Conducting policy analysis and policy briefs in the context of precision nutrition<br>- Reliable sources of information	Learn the essentials of nutrigenetics, nutrigenomics, nutri-epigenetics, and precision nutrition; incorporate nutrigenetic knowledge to policy analysis and policy briefs; identify reliable sources of information and current issues upon which regulations should be discussed	- Development of personalized nutrition guidelines to help reduce the burden of malnutrition.<br>- Identify population subgroups in need of specific dietary interventions.<br>- Complement traditional public health approaches with the nutrigenetic, nutrigenomic and epigenetic-based approach.
**Food Industry**	- Introduction to nutrigenetics, nutrigenomics, nutri-epigenetics, and precision nutrition<br>- Applications of precision nutrition in the food industry and in public health (from policy to action)<br>- Reliable sources of information	Learn the essentials of nutrigenetics, nutrigenomics, nutri-epigenetics, and precision nutrition; knowledge of using precision nutrition in the food industry (i.e., targeting customers in product development and health claims); and identify reliable sources of information	- Utilize data from nutrigenetics, nutrigenomics and nutriepigenetics to develop functional foods and nutraceuticals. Development of specialized diets for individuals or sub-groups of populations with a high genetic risk for malnutrition-related diseases.
**All Stakeholders**	- Forum and workshops: Involvement of all stakeholders to share knowledge, and discuss and solve problems in multidisciplinary teams	Learn to work in multidisciplinary groups to implement precision nutrition	- Reduce the burden of malnutrition-related diseases through the joint effort of stakeholders.
